# Live-Birth Incidence of Isolated D-Transposition of Great Arteries—The Shift in Trends Due to Early Diagnosis

**DOI:** 10.3390/diagnostics14111185

**Published:** 2024-06-05

**Authors:** Andreea Florentina Stancioi-Cismaru, Marina Dinu, Andreea Carp-Veliscu, Razvan Grigoras Capitanescu, Razvan Cosmin Pana, Ovidiu Costinel Sirbu, Florentina Tanase, Florentina Gratiela Dita, Maria Adelina Popa, Mihai Robert Robu, Mihaela Gheonea, Stefania Tudorache

**Affiliations:** 1 Obstetrics and Gynecology Department, Dragasani City Hospital, 245700 Dragasani, Romania; andreea.cismaru23@gmail.com; 2 Doctoral School, University of Medicine and Pharmacy of Craiova, 200349 Craiova, Romania; foriii.manolache@yahoo.com; 3 8th Department, Faculty of Medicine, University of Medicine and Pharmacy of Craiova, 200349 Craiova, Romania; razvan.capitanescu@umfcv.ro (R.G.C.); ovidiusoc@yahoo.com (O.C.S.); mihaela.gheonea@umfcv.ro (M.G.); 4 Department of Obstetrics and Gynecology, Carol Davila University of Medicine and Pharmacy, 020021 Bucharest, Romania; andreea_veliscu@yahoo.com; 5 Panait Sirbu Clinical Hospital of Obstetrics and Gynecology, 060251 Bucharest, Romania; 6 Obstetrics and Gynecology Department, Emergency University County Hospital, 200349 Craiova, Romania; razvancosminpana@yahoo.com (R.C.P.); florentina.tanase@umfcv.ro (F.T.); adelina1popa@gmail.com (M.A.P.); mihai.roberttt@gmail.com (M.R.R.)

**Keywords:** transposition of great arteries, ultrasound, prenatal diagnosis, termination of pregnancy, live-birth incidence, counseling

## Abstract

This is a single tertiary population-based study conducted at a center in southwest Romania. We retrospectively compared data obtained in two periods: January 2008–December 2013 and January 2018–December 2023. The global incidence of the transposition of great arteries in terminated cases, in addition to those resulting in live-born pregnancies, remained almost constant. The live-birth incidence decreased. The median gestational age at diagnosis decreased from 29.3 gestational weeks (mean 25.4) to 13.4 weeks (mean 17.2). The second trimester and the overall detection rate in the prenatal period did not significantly change, but the increase was statistically significant in the first trimester. The proportion of terminated pregnancies in fetuses diagnosed with the transposition of great arteries significantly increased (14.28% to 75%, *p* = 0.019).

## 1. Introduction

The reported prevalence of major congenital heart disease (MCHDs) varies from 0.8% to 1.5% (close to 1%) [1,2]. Dextro-transposition of the great arteries (D-TGA) is the second most common cyanotic congenital heart defect, accounting for 4–5% of all congenital heart defects [3,4], with an incidence of 0.02–0.05% of live births [3]. A 2:1 male predominance has been reported [4].

Although it is a major defect, D-TGA is seen as a surgically correctable disease. The most common surgical procedure used is the Jatene arterial switch operation (ASO) [5], with a perioperative mortality of about 2–4% [6,7,8,9], long-term benefits [7], and a high long-term survival rate [6,10] (10-year survival rate—88–97% [6], 20-year survival rate—nearly 90%) [10]. However, neurodevelopmental dysfunctions are frequently reported (reduced performance in attention, visual–spatial skills, and executive functions, and anxiety disorders) [6,11]. It was hypothesized that delayed brain development in utero heightens postnatal susceptibility to acquired white matter injury [12,13]. These symptoms may be results of TGA per se or results of ASO complications. More studies are needed to confirm if the appropriate perinatal management is enough in isolated D-TGA to reach a good long-term prognosis.

Routine obstetric ultrasound (US) aims to identify suspected cases of CHD so these patients can be referred for full fetal echocardiography. Currently, the question of whether the detection rate (DR) of MCHDs is acceptably high when screening in low-risk pregnancies has remained unanswered. Previously published studies included populations and techniques that differed considerably. Thus, the reported DRs of MCHDs have huge variations. 

TGA is the most representative CHD for the ductal-dependent group. According to some researchers, prenatal diagnosis (PND) is paramount in ductal-dependent CHDs because morbidity and mortality may be reduced [14,15,16]. Yet, PND’s role in reducing mortality and long-term morbidity in TGA (doctors’ awareness, emergent neonatal management strategies, balloon atrial septostomy, short-interval ASO) is still debated [8,10,14,17,18,19]. Thus, it is still necessary to prove the benefits of routine screening and, even more so, those of the diagnosis of TGA in early pregnancy. 

Moreover, in TGA, DRs range widely from 3% [20], 6.9% [21], and 20% “in many first world centers” [22] to 54% [23] and 72% (very high) [24]. Despite advances in fetal echocardiography and technologically impressive improvements, the PND of TGA is reported to be low in general [25,26].

Different approaches to prenatal screening also exist in Romania, and access to routine scans varies within the country due to geographical, social, and economic limitations. We hypothesize that the PND rates for all CHDs vary; thus, this will induce various rates in TOPs. There are no nationwide population-based studies examining the incidence of CHDs or TOPs for CHD in Romania.

Fetal cardiac scanning in the late first-trimester (FT) anomaly scan (FTAS) at 11–13 GW has been introduced progressively in the prenatal diagnosis unit (PDU) of the Emergency University County Hospital (EUCH) of Craiova since 2010, and became mandatory at the end of 2013 [27,28]. The internal policy changed, and simplified cardiac sweep [29,30,31] was attempted universally in every case scanned. Standardized cardiac sweeps were obtained afterward in more than 95% of women. The PDU team comprises six maternal–fetal specialists with experience in the field (more than 20 years), and the hospital benefits from periodic multidisciplinary meetings, including pediatric cardiologists. 

We aimed to evaluate the incidence, DRs, gestational age at diagnosis, and TOP of isolated D-TGA and assess their trends over time in our unit.

## 2. Materials and Methods

We conducted a retrospective study including all newborns with TGA in the EUCH, as well as fetuses diagnosed with TGA leading to TOP, in two different periods: 1 January 2008–31 December 2013 and 1 January 2018–31 December 2023. We aimed for isolated (uncorrected, D-type) TGA cases. We considered “isolated TGA” (iTGA) all cases without major cardiac and extracardiac defects, but we included cases with the common association of ventricular septal defect (VSD). TGA was considered “complex” when associated with any other major cardiac/extracardiac malformations, and these cases were excluded from the analysis. 

Inclusion criteria for cases diagnosed in the second trimester (ST) were typical images of D-TGA [22,32,33,34] (Supplementary Materials: Video S1: ”ST_TGA”) and the absence of associated major cardiac anomalies (other than a small trabecular below 3 mm VSDs). In our view, complete diagnosis is achievable in fetuses scanned later than 22 gestational weeks (GW) only. Yet, for the purpose of this study, we included all cases suspected earlier in pregnancy, if they reached a consensus from at least 3 consultants in maternal–fetal medicine (highly experienced specialists) (Supplementary Materials: Video S2: ”FT_TGA”). 

Inclusion criteria for the postnatally diagnosed cases were relevant clinical features and the description of the neonatal echocardiography, as noted in the file by the pediatric cardiologist, in cases managed peripartum in the EUCH. Pulse oximetry screening was initiated before 2008 and was fully operational in the second period of the study. In the EUCH, neonatal surgery is not available; all cases were treated with prostaglandins, and air transportation was arranged to the Pediatric Intensive Care Unit, Emergency Institute for Cardiovascular Diseases and Transplantation, Targu Mures, Romania (EICVDT). The treatment target saturation before referral was >80% (measured from the right upper arm).

At the first search, nineteen cases met the inclusion criteria and were investigated for inclusion in the study. Four patients were excluded: one had a major associated cardiac anomaly (pulmonary stenosis); one was subsequently diagnosed with a double-outlet right ventricle (DORV); one patient was excluded because the delivery took place in a private foreign institution and the peripartum data were not accessible; and in one case, we found that the US data were not suitable for enrollment.

We included 15 cases: 7 in the historical cohort, and 8 in the recent one. Data were extracted from the patients’ hard-copy and electronic files, the intranet network Hipocrate used by the EUCH of Craiova, as well as the electronic registries of Natalcare (Operational Program Human Capital, Complex Program, Information and Care for Mother and Child—Project Code 142081 (the European Social Fund)) and the Materna clinic. All these registries prospectively record the diagnoses made during outpatient visits and hospital admissions. In the Hipocrate database, a search was undertaken for diagnosis codes Q24–26 and Q20–28 for newborns with a TGA diagnosis in the study period. A separate list of cases was created from the database of patients suspected of TGA in prenatal life. Medical and US files were then reviewed, and relevant data was extracted. This comprised clinical information only, to ensure anonymity and to comply with information governance; no patient identifiers were recorded. All US files of cases resulting in TOPs were reviewed by 3 maternal–fetal specialists. Photographic files of cases with autopsy performed were reviewed. The postoperative outcome was obtained in cases referred for neonatal surgery.

The minimum and maximum follow-up periods for the 8 children with ASO were 2 days and 12 years, respectively. The median follow-up was 45 months (IQR 92).

### 2.1. Study Size

As a local population-based study, this study was limited by the number of fetuses scanned and registered by the institutions involved and by the number of cases of TGA born within the two periods of data collection. If data items were missing from the notes or the US file, cases were excluded from the specific outcome analysis between cohorts.

### 2.2. Approval

This study was conducted according to the guidelines of the Declaration of Helsinki. This is an observational and retrospective study, employing almost exclusively registry research. No patients’ details are included in the manuscript and all patients underwent scans and medical care as part of routine clinical practice and not for research. Therefore, this study did not require registration, approval from the research ethics committee, or informed consent.

### 2.3. Screening Ultrasound (US) Protocol

Ultrasound examinations were performed using highly similar US equipment: Voluson^®^ 730Expert and Voluson E6 and E8 machines (GE Medical Systems Chicago, IL, USA) in the first period, and Voluson E8 and E10 (GE Medical Systems Chicago, IL, USA) US machines in the second. All systems were equipped with volumetric probes: 4–8 MHz curvilinear transducers and 5–9 MHz transvaginal probes.

Nuchal translucency (NT) scanning at 11–13 WG was introduced in our unit in 2008. We used a US scan at this gestational age to establish the risk of aneuploidy using the combined test [35,36,37]. Before implementation, we organized programs to train the operators: we used theoretical knowledge courses, hands-on scanning experience, and certified international online courses.

During this time, the standard prenatal care included a second-trimester (ST) anomaly scan between 18 and 23 WG. Obtaining views of the left and right ventricular outflow tracts (LVOT and RVOT) was attempted as an integral part of the fetal cardiac screening examination in the ST, in both periods of the study [38,39,40]. Although several guidelines were available for fetal echocardiography, in our unit, complying with the International Society of Ultrasound in Obstetrics and Gynecology (ISUOG) guidelines was recommended and followed in both periods [40,41,42,43,44].

A recent publication offers detailed information on basic and advanced fetal echocardiography US scanning, according to current guidelines from all major scientific societies [45].

We performed routinely advanced ST fetal echocardiography [40,42,44] in all cases with US-based indications: increased NT thickness, an abnormal ductus venosus waveform, tricuspid regurgitation during the FT, suspected cardiac structural anomaly, major extracardiac abnormality, chromosomal/genetic anomaly, hydrops, effusions, a single umbilical artery, intrauterine growth restriction, fetal cardiac rhythm abnormalities, and unsatisfactory fetal heart views during routine fetal examinations. In both periods, we used high-frequency transducers, high frame rates (more than 25 frames per second), increased contrast, and high resolution. Low persistence, a single acoustic focal zone, a narrow image field, and magnification were also recommended. The optimal color Doppler settings included the use of a narrow color box and appropriate pulse repetition frequency (PRF) according to the flow velocity of each specific vessel. Low color persistence and adequate gain settings were set to display flow across valves and vessels [45].

**Figure 1:**
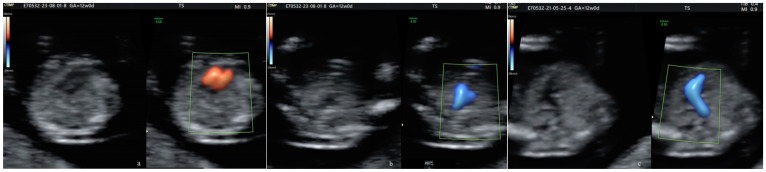
Details at the FT cardiac sweep in a normal (**a**,**b**) and in a TGA case (**c**).

Between 2008 and 2013, prenatal screening for CHDs was not offered routinely at the end of the FT. Since 2013, in line with the general shift to the late FTAS [28,41,46,47,48], the aim of the “nuchal scan” widened, and we targeted all major structural abnormalities. As a major advantage, FTAS was seen as an excellent tool for early reassurance, which was very important in some cases (advanced maternal age, positive history for major structural/chromosomal anomalies, pregnancies after assisted reproductive techniques, and others). Subsequently, the FT fetal cardiac scan became routine. Simplified cardiac sweep [30,31,49,50] was attempted universally in every case scanned at 11–13 WG in our unit (Figure 1). We commonly use the duplex 2D-2D color Doppler. At the FTAS, all ST features recommended for the TGA diagnosis were searched [28,51,52]. When using color Doppler/power Doppler, the mechanical and thermal indices were kept as low as possible (ALARA principle) [53] and the current guidelines were followed [43].

The cardiac sweep at the FTAS contains two planes considered mandatory in the PDU (a and b). The second (the 3VT view plane) is severely distorted in all malposed-aorta cases, and is typical (not pathognomonic) in TGA.

- A normal 4-chamber view and normal equal ventricular inflows in a normal case.
- Confluence of the arches on the left (“the V-sign” in the 3VT view) in a normal case.
- The “reverse boomerang sign”—the reverse curvature of the right ventricle outflow tract (RVOT) at the level of the 3VT view in a TGA case.

**Figure 2:**
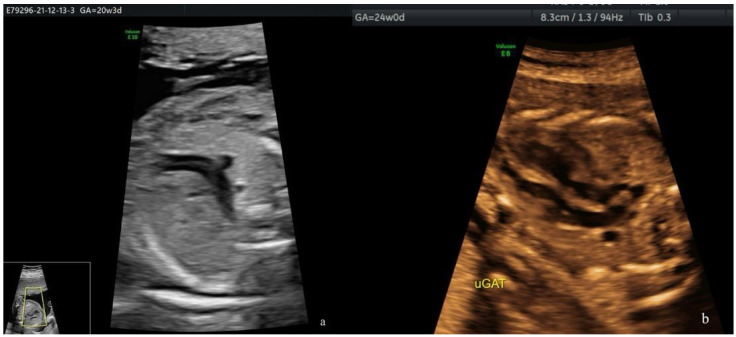
TGA cases: right ventricle outflow tract (RVOT) at level of the three-vessel and trachea (3VT) view (**a**) and parallel arterial trunks (**b**).

Since 2013, all pregnant women cared for in the PDU of the EUCH have been offered at least two anomaly scans: the FTAS (at 11–13 WG) and the STAS (at 18–23 weeks) (Figure 2). The third-trimester (TT) scan remains optional, and is performed at the attending physician’s discretion. Yet, the TT scan was commonly recommended in our unit during both periods assessed. 

We included all cases with suspected or confirmed TGA diagnosis in which a complete consensus about the diagnosis was reached. These cases (resulting in live birth or TOPs, and TOP followed by an informative/documented autopsy or not) were included regardless of the gestational age.

The population scanned in the PDU is generally low-risk and unselected. Yet, approximately 20–22% of cases are referred, due to the tertiary status of the unit. Usually, if any CHD is suspected at US examination in counties surrounding that of our hospital, the patient is referred to a specialist in fetal medicine for fetal echocardiography in our unit. 

All couples with suspicion of TGA were offered genetic counseling and testing, including quantitative fluorescence–polymerase chain reaction (QF-PCR), conventional karyotyping, and array CGH. 

According to internal policy, in our unit, all couples are counseled following a US scan showing abnormal results. The multidisciplinary team composition is customized depending on the respective fetal pathology. All couples entering this study were counseled by a team including a maternal–fetal specialist, a pediatric cardiologist, and a neonatologist. In the second period, a unit psychologist was employed and added to the team, as a certified consultant in prenatal diagnosis.

If the PDU team anticipates the necessity of surgical or complex multidisciplinary treatment shortly after birth, the delivery is set to take place in another tertiary center. Our cases were referred (both prenatally and postnatally) to the most representative public tertiary center for the surgical treatment of CHD in Romania, the EICVDT, Targu Mures. 

### 2.4. Live Births

In the Hipocrat Network, patients who at any time had been given a Q24–26 and Q20–28 code (corresponding to congenital malformations of cardiac chambers, great vessels, cardiac valves, septae, and the venous system) were searched. CHDs were identified. Extracted data included identification number, neonate sex, weight, Apgar score, and all procedural codes given. 

We identified patients with TGA based on the Q25 code. In the EUCH, neonatal surgery is unavailable, so we followed the cases after referral to the EICVDT. Patients’ postnatal US records were examined, and cases with an outcome available for verification postnatally were included. In TGA cases, routine management is performed via continuous pulse oximetry and daily US exams to document the ductus arteriosus’ diameter, and the continuous infusion of prostaglandin E1 (PGE1) to maintain its patency, when needed. After excluding patients with incomplete medical records, we included 8 pregnancies ending at term, among which 3 cases were diagnosed postnatally. We referred all cases (5 were transported in utero, and 3 after birth).

One newborn died before emergency intervention was possible, and one newborn was lost immediately after the Raskind procedure. All 6 remaining babies were subjected to ASO, at intervals between 2 days and 8 days. Among these, 2 were genetically tested, and the molecular karyotype showed no significant changes. They all evolved uneventfully after surgery, but 3 of them had suboptimal long-term neurodevelopment. No differences in long-term outcomes were identified between the children diagnosed prenatally and the ones diagnosed postnatally.

### 2.5. Termination of Pregnancy

We searched for O04.0–O04.5 and O35.8–O35.9 codes related to TOP for congenital abnormalities. We searched for all iatrogenic TOPs after 12 weeks of gestation and selected for inclusion the ones diagnosed with TGA. We included the mother’s identification number, place, gestational age, and date of the TOP. We reviewed the records and the autopsy files following TOPs. TOPs not followed by autopsy or followed by non-informative autopsy were not excluded from the study.

### 2.6. Analyses

Rates were calculated as follows:

- Incidence of TGA was calculated as the number of TGA cases (live-born and terminated) divided by the total number of fetuses scanned.
- Live-birth incidence of TGA was calculated as the number of TGA cases in live-born children divided by the total number of live births.

DRs for TGA were calculated as the number of prenatally diagnosed TGA cases divided by the total number of fetuses (live-born and terminated) with TGA. The TOP rates were calculated as the number of terminated fetuses with TGA divided by the total number of fetuses with TGA (live-born and terminated). 

Categorical data are presented as frequencies and percentages along with their respective 95% confidence intervals. Differences in proportions were evaluated using z-tests, and logistic regression was employed to investigate the influence of factors such as BMI on the PND of TGA. The analyses were performed using Python the 3.11.5 version, which facilitated data manipulation and the calculation of confidence intervals and *p*-values, and IBM SPSS Statistics, version 26, for logistic regression modeling. All hypothesis testing was two-sided, and a *p* value of less than or equal to 0.05 was considered indicative of statistical significance.

## 3. Results

**Figure 3:**
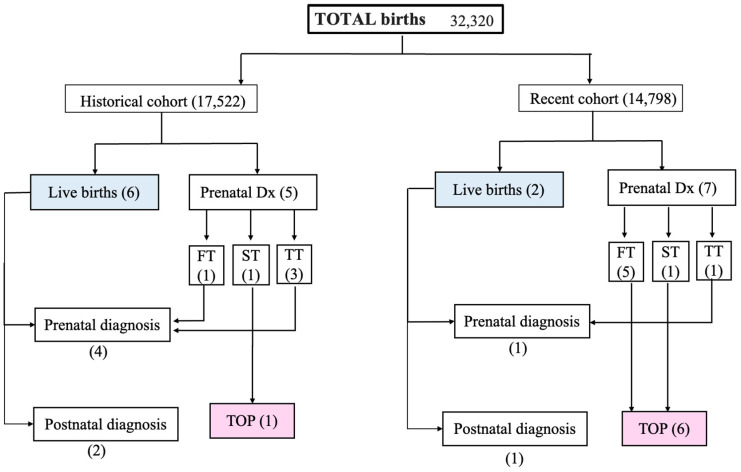
Flowchart presenting the selected study population and the number of cases in both cohorts. Abbreviations: Dx = diagnosis; FT = first trimester, ST = second trimester, TT = third trimester; TOP = termination of pregnancy.

A flow chart is shown in Figure 3.

In total, we had 32320 births at our institution during the two intervals selected for this study. Five fetuses were referred to be born at the EICVDT, and three neonates were referred postnatally.

In total, 13 fetuses with TGA were identified in prenatal life and we had 7 terminated pregnancies after 12 weeks of gestation. Six of them belonged to the recent cohort.

### 3.1. Incidence

During the first period of the study, 17,522 children were born, resulting in a live-birth incidence of TGA of 0.034% (95% CI = 0.00685–0.06164%). During the second period of the study, 14798 children were born, resulting in a live-birth incidence of TGA of 0.013% (95% CI = 0.000–0.03225%).

This does not represent a significant change when comparing the two periods throughout the study (*p* = 0.238). 

The live-birth incidence of TGA decreased from 0.034% to 0.013% (OR = 0.395, 95% CI = 0.080–1.1955; *p* = 0.238).

An influence of general prenatal screening was not confirmed (OR = 2.8, 95% CI = 0.196–40.059, *p* = 0.438).

In our case series, TGA was not significantly more common in males than in females (OR = 1.5, 95% CI = 0.353–0.812, *p* = 0.273).

When including TOPs, the incidence of TGA in all fetuses was 0.062% (95% CI = 0.0104–0.0695%) during the first period and 0.060% (95% CI = 0.0166–0.0915%) during the second, with no significant changes over time.

### 3.2. Detection Rates

**Figure 4:**
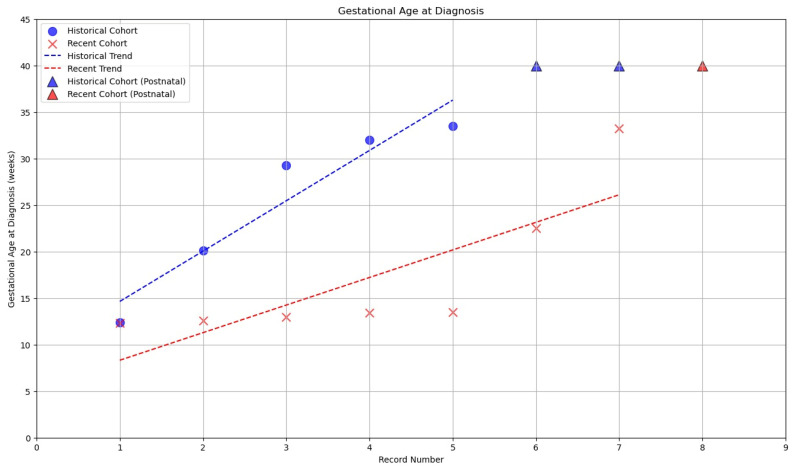
Graphical representation of gestational age at diagnosis (blue dots—the historical cohort, pink dots—the recent cohort; trends are represented by dotted lines).

The individual gestational age at diagnosis of TGA is shown in Figure 4. 

During the two study periods, 12/15 (80%) fetuses with TGA (95% CI = 0.53–0.94) were diagnosed prenatally, without sex-specific differences. The overall increase in DRs was not statistically significant when comparing the two periods (71.42% vs. 87.5%) (*p* = 0.438). 

The increase was statistically significant in the FT (*p* = 0.05) and did not reach statistical significance in the ST (OR = 1.5; 95% CI = 0.071–31.575; *p* = 0.793).

In the historical cohort, one case only was diagnosed in the FT, whereas in the third trimester (TT), 42.85% cases were detected. 

In the second period of the study, there was a significant difference in detection rates between the first and second trimesters (71.43% vs. 14.29%; *p* = 0.031). 

In the prenatally diagnosed group (PND+ group) of 12 patients, TGA was diagnosed at a median gestational age of 29.3 gestational weeks (mean 25.4) in the historical cohort, and at a median of 13.4 weeks (mean 17.2) in the recent one. 

In the global population studied, TGA was diagnosed prenatally at an average of 20.65 weeks GA, with 50% diagnosed during the FT, 17% in the ST, and 33% in the TT.

There was no significant difference in DRs considering the type of practice, whether public or private (*p* = 0.736).

Body mass index (BMI) did not influence the PND of TGA. The BMI coefficient is negative (B = −0.489), but its *p*-value is 0.068, which is above the conventional threshold for significance (0.05). Thus, higher BMI is associated with a lower likelihood of PND+, but this result is not statistically significant. There was a suggestive but not statistically significant increased risk of missed PND for overweight and obese patients (BMI > 25 kg/m^2^).

### 3.3. Characteristics of Live-born TGA at the EUCH 

The timing of the diagnosis of TGA in live-born fetuses included in this study is presented in Table 1. The differences between the two cohorts are highlighted in the *p* Value column.

### 3.4. Termination of Pregnancy

During the study periods, 7/15 (46.66%) (CI 95% = 0.73–55.42%) of all pregnancies with TGA were iatrogenically terminated, on parental request. In other words, 7 cases ended in TOPs among the 12 cases diagnosed prenatally (58.33%). In the two cohorts presented, all TOPs were medically performed; we had no surgically terminated cases. 

**Figure 5:**
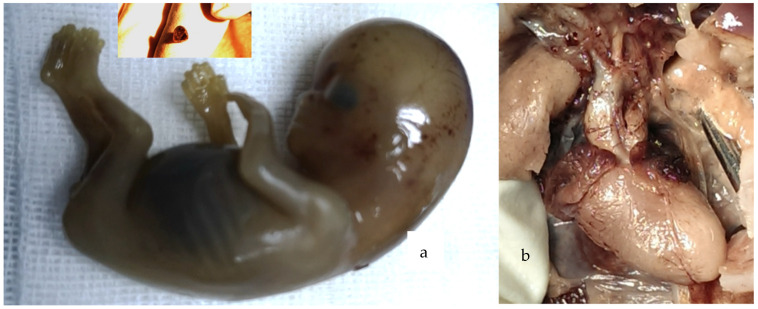
Postabortum conventional autopsy details: (**a**) Specimen after first-trimester medical TOP; CRL (crown–rump length) 68 mm. In upper window, fetal heart is shown in transverse section on operator’s fingers; comparative size can be seen. (**b**) Specimen after second-trimester medical TOP; an accurate diagnosis of TGA is reachable.

Autopsy was performed in both fetuses terminated in the ST. The autopsy files were found to be relevant. Although intact specimens were obtained in four out of five fetuses terminated after the FT diagnosis, informative images from the fetal conventional autopsy were lacking (Figure 5), and we had one case confirmed postmortem using a non-conventional method. 

For pregnancies with prenatally diagnosed TGA, the proportion of TOPs changed over time. 

TOPs increased from one case in seven (14.29%) in the historical cohort to six in eight (75%) (CI 95% = 36.46%–95.36%) in the recent cohort (*p* = 0.0187)

## 4. Discussion

This is the first study to describe the time trends in the incidence, detection rates, and TOP rates for iTGA in a tertiary center in Romania. Two periods separated by a 10-year interval are presented. 

Of 256 neonates and fetuses diagnosed with congenital heart and great vessel abnormalities, 213 had major congenital heart diseases (MCHDs). The overall incidence was 0.79% for all CHD cases and 0.04% for TGA cases. These figures are in line with most estimates [1,54,55,56,57]. There are controversies about the time trends of the incidence of CHD [58,59]. We did not find significant changes over time in the overall incidence of MCHD or TGA. 

We chose iTGA for our investigation due to case homogeneity, and due to the reported excellent postsurgical prognosis among ductal-dependent CHDs. Moreover, this critical CHD has a notorious negative correlation with chromosomal abnormalities [60]; thus, we considered this specific condition the most worthwhile for searching.

The main findings of this study are that while the general incidence of TGA was stationary, the live-birth incidence decreased significantly—by 66.66%. Furthermore, comparing the two periods, we found a 5-fold increase in TOP rates. Similar results, showing that the decrease in the live-birth incidence of MCHD follows an increased TOP rate, were previously reported for a univentricular heart [61].

Generalizing cardiac sweep is both ethical and rational, as it is known that most CHD occurs in pregnancies with no risk factors [62], and all parents that choose to be tested by US scan have the right to find out as early as possible about suspected structural abnormalities [63,64].

It was previously found that TOP rates for structural abnormalities are higher in fetuses diagnosed in the FT compared with those diagnosed in the ST [65]. In our study, an increased TOP rate was also associated with higher DRs of TGA prenatally, especially in early pregnancy. 

In the second period of the study, DRs were higher in both the FT and the ST; these results are consistent with other recently conducted studies [66,67]. This may be explained by the increase in doctors’ skills and awareness and may be partially attributed to the developments in obstetric US equipment. Also, the change in internal policy, with the introduction of routine cardiac sweep (the implementation of the three-vessel and trachea view) at the FTAS and the sustained effort to implement published guidelines, may be considered.

Most probably, it is not feasible to reach a 100% detection rate of TGA. Yet, in our recent cohort, all fetuses presented in the unit for FTAS and for STAS were detected, with the exception of one (seven out of eight). Characteristic US features were present in all fetuses. The missed case was related to a relevant scan, and iTGA should have been readily detected. Unfortunately, the images were misinterpreted by a less experienced operator. Our findings, as well of those of others [2], confirm that the clinical experience of the sonographer influences prenatal detection.

In our case series, all three cases diagnosed postnatally had US examinations during pregnancy in lower-ranked hospitals in neighboring counties. It is desirable to ensure training and adequate examinations across hospitals. The complete implementation of 3-VTv and outflow tract views enhances the detectability of iTGA, but further centralization of malformation scans may improve DRs.

The prenatal diagnosis of any major CHD always raises the question of whether to terminate or to continue the pregnancy, and this decision will depend on a variety of factors [24,68,69]. 

Currently, TOP is performed in 50–60% of all fetuses with MCHD diagnosed prenatally [70]. TOPs for TGA also vary largely from roughly 12 [55] to 21% [70] and to 72% [71]. In certain countries, a decrease in TOPs of TGA is registered [70].

The ethics of TOPs in TGA are beyond the aim of this manuscript. But it is worth mentioning that in Romania, TOP at the parents’ request is regulated until 14 weeks GA [72]. If major or suboptimally repaired congenital abnormalities are detected later, it is customary that an ethics board is gathered, to adjudge the opportunity to continue the pregnancy. Medically indicated terminations are permitted until 24 weeks GA.

We must highlight that TGA cases (such as tetralogy of Fallot, pulmonary atresia, interrupted aortic arch, coarctation of aorta, and anomalous pulmonary venous return) can be repaired in a biventricular manner. Improvements in management, BAS [73], and ASO procedures have dramatically altered the outcomes for TGA neonates. Currently, the 25-year postoperative survival rate after arterial switch surpasses 96% [74]. Yet, the reported 1-year mortality after ASO of less than 5% [75] can have a huge emotional impact on parents.

In our case series, optimism in multidisciplinary counselling following an FT scan was often futile. Although the team struggled to offer scientifically accurate data, it rarely influenced the parents’ decision to terminate a fetus perceived as severely affected.

All members in the counselling teams highlighted the high chances of a good neonatal outcome following surgery, and the amount of information becoming accessible by US later in pregnancy.

The limited access to high-quality neonatal surgery and difficulties faced by parents engaged in the long-term care of a child born with TGA may also be considered.

In the longer term, maintained increased TOP rates will lead to a decreased live-birth incidence of TGA and, subsequently, to lower patient flow in tertiary centers with neonatal surgery availability. This again underlines the importance of PND and raises the discussion of centralization (even for more than one country) for surgical treatment. Eventually, we may need to find a balance between the requirement for high levels of procedural experience, crucial for the invasive treatment of TGA, and the longer distances needed for in utero/neonatal transportation.

In our view, the current performance in FT prenatal screening still implies two important disadvantages: uncertain results in estimating actual postnatal severity and the risk of TOP at the request of parents. In the latter group, an accurate follow-up diagnostic is absent due to fetal size restriction [76], not widely accepted, or difficult to perform [77,78,79]. Conversely, an ST scan is more accurate and safer, though it has the disadvantage of a long waiting time. 

The counseling after a US screening scan should remain evidence-based and non-directive. Yet, parents should be advised in a respectful, considerate, and empathic manner, to postpone the termination of fetuses with suspected TGA, at least util the US technology is able to provide a complete prenatal diagnosis. Couples should be offered follow-up US scans without the fear of losing a narrow window of opportunity due to law issues.

A similar shift in trends is to be expected in other congenital pathologies: the DRs will increase, due to both technological improvement and the current guidelines [43]. For example, in open spina bifida, high suspicion may already be achieved extremely early in pregnancy [80], yet the accurate location/height, the dimensions, and the structure of the spinal defect remain fully unknown at the FTAS. Thus, counseling will be incomplete or incorrect at the end of the FT. In other highly evolving fetal systems, counselling following the FTAS will also remain suboptimal [49,81].

In all early-diagnosed cases, the parents will face difficult dilemmas. Moreover, the professionals’ tasks will also be increasingly difficult because they will struggle to weigh up different ethical principles: the autonomy of the parents on one hand, and the principles of beneficence, nonmaleficence, and justice on the other. An impaired neonate leads to moral distress and post-traumatic stress reactions in parents. Thus, at times, physicians are tempted to exercise defensive medicine and allow easier incompletely justified medical TOPs. 

The trend we found of increased FT terminations is somewhat concerning. We stress again our study size, which is suboptimal to draw definite conclusions. These worrisome results should be reassessed in larger studies.

To conclude our study, parents with good prognosis fetuses, with correctable (in a biventricular manner) fetal heart abnormalities should be encouraged to continue pregnancy toward the ST. In our view, the ST professional scan, performed by experts in the field, is the key to a more accurate diagnosis and, therefore, to better counseling. To address this issue, we suggest improving access to genetic molecular testing and enhancing support services for families facing prenatal diagnoses. Joining (national or international) support groups specific to TGA can connect families with others having similar experiences and provide emotional support. Therapists or counselors can help parents to cope with the emotional impact of the TGA diagnosis and provide strategies for managing stress and anxiety.

With neonatal cardiac surgery not available at the ECUH, we present a rather homogenous cohort, identifying cases from a geographically defined population, opposite to a population of fetuses/neonates referred to a single tertiary center committed to treating and operating on TGA cases. To some extent, the results of this study are descriptive of both the advances in PND and the limits of surgical correction in our country.

This study has several important limitations. 

The paramount ones are related to active management following the provision of FT scan information, ending in TOPs. This limited both US and pathological investigation. All predictors for urgent BAS and long-term outcomes are still controversial [8,82,83,84], and the most important ones for neonatal outcomes are reachable only in later pregnancy: VSD’s presence/dimensions, the foramen ovale (FO) [85,86,87], ductus arteriosus [87,88], and other findings [89,90] cannot be assessed in early pregnancy. Although favorable counseling at diagnosis remains appropriate, caution should be exercised by counselors. Many cardiac parameters may change late in pregnancy, and neonatal death may occur despite adequate management [87]. 

Also, the coronary anatomy [91,92], a key determinant of both short- and long-term outcomes following ASO, was not investigated in our study [93,94,95]. 

Additionally, we may hypothesize that some of the cases diagnosed in the FT had either an incomplete or a wrong diagnosis [96,97]. Still, we involved all specialists in maternal–fetal medicine in difficult-to-interpret cases, and included only cases that reached a definitive consensus.

In a recent systematic review and metanalysis [98], associations were found between high DR and structured anatomical assessment, the latter improving sensitivity. According to this research, the FT scan yielded similar figures for DRs to the ST scan (over half of fetuses affected by major cardiac pathology). Recently, the most important professional society in prenatal US published GLs supporting this vision [43]. We previously published [27,30,99] our standardized technique to assess the FT heart, by adding visualization of the outflow tracts and color-flow Doppler imaging to the 4CV assessment.

In this study, not all fetuses were genetically tested, although chromosomal microarray analysis (arrayCGH) is seen as a valuable tool to detect chromosomal copy number variation in TGA and may improve prenatal counseling [3,100,101]. In our case series, all parents with suspicion/a diagnosis of TGA at the end of the FT declined the invasive procedure after counseling. We tested two fetuses using amniocentesis and conventional karyotyping, and two neonates were tested by means of arrayCGH. As expected, no significant changes were found.

Miscarriages were not included in this study. Therefore, we may have missed some fetuses with iTGA.

Surgical and follow-up data could not be obtained for one neonate who was not operated on at the EICVDT, which could limit the interpretation of the long-term outcomes.

The relatively small number of patients included hampered any demonstration of the effect on long-term outcomes. According to the published literature, the rates of adverse neurodevelopmental outcomes in children with TGA disease remain relatively high. Further investigation is needed to understand whether antenatal diagnosis has the potential to improve longer-term outcomes [102].

## 5. Conclusions

This study, based on a geographically defined population, showed that the incidence of fetuses with TGA did not change significantly from 2008–2013 to 2018–2023. The prenatal detection of TGA increased, currently approximating 80% in our unit. The gestational age at diagnosis significantly decreased. Simultaneously, overall terminations of pregnancy in TGA reached 85.7% of all fetuses diagnosed prenatally in our case series. The TOP rates increased from 14.28% to 75% (*p* = 0.019 < 0.05) and we registered a subsequent 0.327% decrease in the live-birth incidence of TGA. The trend highlighted by this study may be related to the introduction of routine FT fetal heart scanning in prenatal screening in our unit in 2013. These results may be influenced by cultural, legal, and health system factors; therefore, they may not be generalizable and should be reassessed in larger studies.

The centralization of anomaly scans, training, and technological advances, in addition to relatively easy early diagnosis, will most probably further improve prenatal detection rates in TGA and other major structural abnormalities. Multicentric studies are needed to prevent early and/or unnecessary terminations in cases with a favorable prognosis.

## Figures and Tables

**Table 1:** Characteristics of live-born TGA at the EUCH.

	Prenatal vs. Postnatal Diagnosis, No./Total No. (%)		*p* Value
	2008–2013 (No Universal FT Screening)	2018–2023 (Universal FT Screening)	
TGA FT	1/7 (14.28%)	5/8 (62.5%)	0.34
TGA ST	1/7 (14.28%)	1/8 (12.5%)	1.0
TGA TT	3/7 (42.85%)	1/8 (12.5%)	0.58
Prenatal Dx TGA overall	4/7 (57.14%)	7/8 (87.5%)	0.7
Postnatal Dx TGA	2/7 (28.57%)	1/8 (12.5%)	1.0

Abbreviations: vs. = versus, No = number, FT = first trimester, TGA = transposition of great arteries, ST = second trimester, Dx = diagnosis.

## Data Availability

All ultrasound files, neonates’ files, and autopsy files are available and will be provided upon reasonable request.

## Supplementary Materials

The following supporting information can be downloaded at: https://www.mdpi.com/article/10.3390/diagnostics14111185/s1, Video S1: ST_TGA, Video S2: FT_TGA.
